# Association of melatonin membrane receptor 1A/1B gene polymorphisms with the occurrence and metastasis of hepatocellular carcinoma

**DOI:** 10.18632/oncotarget.21107

**Published:** 2017-09-20

**Authors:** Shih-Chi Su, Yung-Chuan Ho, Yu-Fan Liu, Russel J. Reiter, Chia-Hsuan Chou, Chia-Ming Yeh, Hsiang-Lin Lee, Wen-Hung Chung, Ming-Ju Hsieh, Shun-Fa Yang

**Affiliations:** ^1^ Whole-Genome Research Core Laboratory of Human Diseases, Chang Gung Memorial Hospital, Keelung, Taiwan; ^2^ Department of Dermatology, Drug Hypersensitivity Clinical and Research Center, Chang Gung Memorial Hospital, Taipei, Linkou and Keelung, Taiwan; ^3^ School of Medical Applied Chemistry, Chung Shan Medical University, Taichung, Taiwan; ^4^ Department of Biomedical Sciences, College of Medicine Sciences and Technology, Chung Shan Medical University, Taichung, Taiwan; ^5^ Department of Cellular and Structural Biology, The University of Texas Health Science Center, San Antonio, TX, USA; ^6^ Institute of Medicine, Chung Shan Medical University, Taichung, Taiwan; ^7^ Deptartment of Surgery, Chung Shan Medical University Hospital, Taichung, Taiwan; ^8^ College of Medicine, Chang Gung University, Taoyuan, Taiwan; ^9^ Cancer Research Center, Changhua Christian Hospital, Changhua, Taiwan; ^10^ Graduate Institute of Biomedical Sciences, China Medical University, Taichung, Taiwan; ^11^ Department of Medical Research, Chung Shan Medical University Hospital, Taichung, Taiwan

**Keywords:** melatonin receptor, polymorphism, hepatocellular carcinoma, metastasis

## Abstract

Hepatocellular carcinoma (HCC) is a prevalent primary neoplasm of the liver, whose heterogeneous global incidence suggests the likely impact of genetic variations among individuals on the susceptibility to this disease. Increasing evidence indicates that melatonin exhibits oncostatic properties in many cancer types at least in part mediated by its membrane-bound receptors, melatonin receptor 1A (encoded by *MTNR1A*) and 1B (*MTNR1B*). In this study, the effect of melatonin receptor gene polymorphisms on the risk and progression of hepatic tumors was evaluated between 335 HCC patients and 1196 cancer-free subjects. We detected a significant association of MTNR1A single nucleotide polymorphism (SNP), rs6553010, with the elevated risk of HCC (AOR, 1.587; 95% CI, 1.053–2.389; *p* = 0.027) after being adjusted for two potential confounders, age and alcohol use. In addition, patients who carry at least one polymorphic allele (heterozygote or homozygote) of MTNR1A rs2119882 or rs2375801 were more prone to develop distant metastasis (OR, 5.202; 95% CI, 1.163–23.270; *p* = 0.031, and OR, 7.782; 95% CI, 1.015–59.663; *p* = 0.048, for rs2119882 and rs2375801, respectively). Further analyses revealed that rs2119882 is located on the consensus binding site of GATA2 transcription factor within the promoter region of *MTNR1A* gene, and that a correlation between the levels of GATA2 and melatonin receptor 1A was observed in the TCGA (The Cancer Genome Atlas) dataset. Moreover, individuals bearing a specific haplotype of four *MTNR1B* SNPs were more prone to develop HCC. In conclusion, our data suggest an association of melatonin receptor gene polymorphisms with the risk of HCC and hepatic cancer metastasis.

## INTRODUCTION

Hepatocellular carcinoma (HCC) is currently the sixth most frequent type of cancer with a high mortality and an increasing occurrence worldwide [1]. A huge variation in the incidence of HCC was observed across different geographic regions, with the highest rates in Southeast Asia and Sub-Saharan Africa [2]. Although a majority of HCC (approximately 70–90%) occurs in patients with underlying chronic liver diseases [3], hepatocarcinogenesis is a complex process that is attributed to multiple risk parameters, including but not limited to exposure of aflatoxin B, chronic infection with hepatitis B virus (HBV) or hepatitis C virus (HCV), excessive consumption of alcohol and tobacco, iron overload, and diabetes [4, 5]. Currently, mounting evidence has demonstrated that single-nucleotide polymorphisms (SNPs) are correlated with the development and progression of liver cancer separately or in combination with well-documented risk factors in defined ethnic groups [6–8]. These data pinpoint a key role for genetic polymorphisms that affect oxidative stress, DNA repair, iron metabolism, cell signaling, inflammatory and immune responses in the susceptibility to liver neoplasms and partly account for the global heterogeneous incidence of HCC.

Melatonin (N-acetyl-5-methoxytrypamine), originally isolated as a neurohormone of the pineal grand [9], is synthesized in a myriad of tissue types, including the liver [10]. In the pineal gland, its production is regulated by light and dark cycle, whereby light suppresses and darkness enhances its synthesis. Melatonin controls various biological activities, among which regulation of the phasing of circadian rhythms and sleep promotion are most widely recognized [11]. In addition, this multitasking hormone is known to function as not only a substantial immunomodulatory compound [12] but also a potent free radical scavenger [13]. By virtue of these multiple actions, numerous lines of evidence have linked the perturbation of melatonin signaling to a large number of physiological and pathological conditions including aging, metabolic syndrome, diabetes, immune diseases, hypertension, several mood and cognitive disorders, and cancer [14, 15]. Under both *in vitro* and *in vivo* circumstances, melatonin, employing multiple and interrelated mechanisms, exhibits many oncostatic properties in a variety of tumors during different stages of cancer progression [16–19]. As such, the use of melatonin has emerged as another appealing option for anti-cancer treatment [20].

The broad spectrum of melatonin’s actions are in part mediated by G protein-coupled membrane receptors, melatonin receptor 1A (MT_1_, encoded by *MTNR1A*) and 1B (MT_2_, *MTNR1B*), expressed in both the central nervous system and in numerous peripheral tissues [11]. Genetic associations of *MTNR1A* and *MTNR1B* with several conditions have been documented. Polymorphisms within the *MTNR1B* gene confer a genetic predisposition to type 2 diabetes [21–23], gestational diabetes [24] and adolescent idiopathic scoliosis [25]. Another study has implicated *MTNR1A* gene variations as a risk component in polycystic ovary syndrome [26]. However, little is known regarding the melatonin receptor gene polymorphisms on the susceptibility to HCC. Here, we performed a hypothesis-driven case-control study to evaluate the impact of gene variations of *MTNR1A* and *MTNR1B* on the risk and progression of HCC and observed an association of melatonin receptor gene polymorphisms with the risk of HCC, liver cancer metastasis, and augmented liver damage.

## RESULTS

### Characteristics of study participants

Since age, gender, alcohol consumption, and tobacco use were identified as risk factors for occurrence of liver cancer [27, 28], the demographic parameters between 335 patients with HCC and 1196 normal controls were compared (Table 1). The ratio of males to females in the control group was compatible (*p =* 0.577) with that of HCC patients. The average age of patients at onset of HCC in this study is 62.8 ± 11.7. No significant difference in the distribution of smoking (*p =* 0.740) was achieved between healthy control subjects and HCC patients; however, infections with HBV or HCV and alcohol consumption were found to leverage the risk of developing HCC.

**Table 1:** The distributions of demographical characteristics in 1196 controls and 335 patients with HCC

Variable	Controls (*N* = 1196)	Patients (*N* = 335)	*p* value
Age (yrs)	Mean ± S.D.	Mean ± S.D.	
	59.42 ± 7.08	62.76 ± 11.68	*p <* 0.001*
Gender			
No	358 (29.9%)	95 (28.4%)	*p =* 0.577
Yes	838 (70.1%)	240 (71.6%)	
Cigarette smoking			
No	726 (60.7%)	200 (59.7%)	*p =* 0.740
Yes	470 (39.3%)	135 (40.3%)	
Alcohol drinking			
No	1027 (85.9%)	211 (63.0%)	*p <* 0.001*
Yes	169 (14.1%)	124 (37.0%)	
HBsAg			
Negative	1050 (87.8%)	195 (58.2%)	*p <* 0.001*
Positive	146 (12.2%)	140 (41.8%)	
Anti-HCV			
Negative	1143 (95.6%)	181 (54.0%)	*p <* 0.001*
Positive	53 (4.4%)	154 (46.0%)	
Stage			
I+II		223 (66.6%)	
III+IV		112 (33.4%)	
Tumor T status			
T1+T2		225 (67.2%)	
T3+T4		110 (32.8%)	
Lymph node status			
N0		326 (97.3%)	
N1+N2+N3		9 (2.7%)	
Metastasis			
M0		319 (95.2%)	
M1		16 (4.8%)	
Child-Pugh grade			
0 or A		260 77.6%)	
B or C		75 (22.4%)	
Liver cirrhosis			
Negative		61 (18.2%)	
Positive		274 (81.8%)	

Mann-Whitney *U* test or Fisher’s exact test was used between healthy controls and patients with oral cancer. *p value < 0.05 as statistically significant.

Child-Pugh grade: 0 or A: scores < 7; B: scores = 7–9; C: scores > 9.

### Association of melatonin receptor gene polymorphisms with HCC

Many oncostatic properties of melatonin have been demonstrated in different stages of cancer development [17, 18, 29], yet a correlation of melatonin receptor gene polymorphisms with the risk and progression of liver cancer remains undefined. To address this, five *MTNR1A* SNPs (rs13140012, rs6553010, rs2119882, rs13113549, and rs2375801) as well as five *MTNR1B* SNPs (rs1387153, rs1562444, rs4611171, rs10765576, and rs10830963) were evaluated in this investigation. Genotype frequencies of these gene polymorphisms and their association with the susceptibility to liver cancer are shown in Table 2. No deviation (*p* > 0.05) from Hardy-Weinberg equilibrium in either study group was achieved for all SNPs. To lower the potential interference of other confounders, AOR (with 95 % CI), which was assessed by multiple logistic regression models after adjustment for age and alcohol use, was used together with OR (with 95 % CI) in each comparison. Among the gene polymorphisms tested, homozygotes (GG) for the minor allele of *MTNR1A* rs6553010 were marginally more prone to develop HCC with the OR being 1.445 (95% CI, 0.980–2.132; *p =* 0.063). While adjusted for age and alcohol use, a significant association of the homozygous phenotype (GG) for the minor allele of rs6553010 with the predisposition to HCC (AOR, 1.587; 95% CI, 1.053–2.389; *p =* 0.027) was detected. Nevertheless, no difference in genotype frequencies for the other variants of *MTNR1A-MTNR1B* gene individually between the two cohorts was observed.

**Table 2:** Genotyping and allele frequency of *MTNR1A-MTNR1B* single nucleotide polymorphism (SNP) in HCC and normal controls

Variable	Controls *N* = 1196 (%)	Patients *N* = 335 (%)	OR (95% CI)	AOR (95% CI)
**rs13140012**				
AA	490 (41.0%)	130 (38.8%)	1.000 (reference)	1.000 (reference)
AT	557 (46.6%)	164 (49.0%)	1.110 (0.856–1.440)	1.099 (0.837–1.444)
TT	149 (12.4%)	41 (12.2%)	1.037 (0.698–1.541)	1.087 (0.719–1.645)
**rs6553010**				
AA	553 (46.2%)	140 (41.8%)	1.000 (reference)	1.000 (reference)
AG	520 (43.5%)	150 (44.8%)	1.139 (0.879–1.477)	1.191 (0.907–1.564)
GG	123 (10.3%)	45 (13.4%)	1.445 (0.980–2.132)	1.587 (1.053–2.389)*
**rs211988**2				
TT	482 (40.3%)	138 (41.2%)	1.000 (reference)	1.000 (reference)
TC	570 (47.7%)	158 (47.2%)	0.968 (0.748–1.254)	0.960 (0.732–1.260)
CC	144 (12.0%)	39 (11.6%)	0.946 (0.633–1.413)	1.070 (0.704–1.626)
**rs13113549**				
GG	475 (39.7%)	133 (39.7%)	1.000 (reference)	1.000 (reference)
GA	566 (47.3%)	162 (48.4%)	1.022 (0.788–1.325)	1.014 (0.772–1.331)
AA	155 (13.0%)	40 (11.9%)	0.922 (0.620–1.371)	0.961 (0.634–1.455)
**rs2375801**				
AA	401 (33.5%)	110 (32.8%)	1.000 (reference)	1.000 (reference)
AG	603 (50.4%)	171 (51.0%)	1.034 (0.788–1.355)	1.057 (0.795–1.404)
GG	192 (16.1%)	54 (16.2%)	1.025 (0.709–1.482)	1.111 (0.755–1.636)
**rs1387153**				
CC	342 (28.6%)	98 (29.3%)	1.000 (reference)	1.000 (reference)
CT	601 (50.3%)	160 (47.8%)	0.929 (0.699–1.235)	0.877 (0.650–1.182)
TT	253 (21.1%)	77 (22.9%)	1.062 (0.756–1.492)	1.034 (0.724–1.476)
**rs1562444**				
AA	570 (47.7%)	156 (46.6%)	1.000 (reference)	1.000 (reference)
AG	512 (42.8%)	146 (43.6%)	1.042 (0.807–1.345)	1.046 (0.800–1.367)
GG	114 (9.5%)	33 (9.8%)	1.058 (0.691–1.620)	1.078 (0.691–1.681)
**rs4611171**				
GG	578 (48.3%)	160 (47.8%)	1.000 (reference)	1.000 (reference)
GT	501 (41.9%)	146 (43.3%)	1.046 (0.811–1.349)	1.049 (0.803–1.371)
TT	117 (9.8%)	30 (8.9%)	0.926 (0.598–1.435)	0.935 (0.592–1.475)
**rs10765576**				
GG	579 (48.4%)	166 (49.6%)	1.000 (reference)	1.000 (reference)
GA	511 (42.7%)	136 (40.6%)	0.928 (0.719–1.199)	0.934 (0.714–1.222)
AA	106 (8.9%)	33 (9.8%)	1.086 (0.708–1.664)	1.081 (0.692–1.690)
**rs10830963**				
CC	360 (30.1%)	103 (30.8%)	1.000 (reference)	1.000 (reference)
CG	611 (51.1%)	162 (48.4%)	0.927 (0.701–1.225)	0.838 (0.625–1.123)
GG	225 (18.8%)	70 (20.8%)	1.087 (0.769–1.537)	1.045 (0.727–1.502)

The adjusted odds ratios (AORs) with their 95% confidence intervals (CIs) were estimated by multiple logistic regression models after controlling for age and alcohol drinking. *p < 0.05 was considered statistically significant.

### Correlation between polymorphic genotypes of MTNR1A-MTNR1B and clinical status of HCC

Since rs6553010 is associated with the risk of HCC, the correlations of the melatonin receptor gene polymorphisms with clinicopathologic characteristics of HCC patients were further explored. We found that instead of rs6553010, patients who carry at least one polymorphic allele (heterozygote or homozygote for the minor allele) of *MTNR1A* rs2119882 (Table 3) or rs2375801 (Table 4) were more inclined to develop distant metastasis (OR, 5.202; 95% CI, 1.163–23.270; *p =* 0.031, and OR, 7.782; 95% CI, 1.015–59.663; *p =* 0.048, for rs2119882 and rs2375801, respectively). However, none of *MTNR1B* SNPs examined was found to be individually associated with clinical stage, tumor size, lymph node metastasis, distant metastasis, vascular invasion, Child-Pugh classification, prevalence of hepatitis B virus (HBV) and hepatitis C virus (HCV) infections, and cirrhosis.

**Table 3:** Odds ratio (OR) and 95% confidence interval (CI) of clinical status and *MTNR1A* rs2119882 genotypic frequencies in 335 HCC patients

Variable	Genotypic frequencies			
	TT (*N* = 138)	TC+CC (*N* = 197)	OR (95% CI)	*p* value
Clinical Stage				
Stage I/II	88 (63.8%)	135 (68.5%)	1.00	*p =* 0.364
Stage III/IV	50 (36.2%)	62 (31.5%)	0.808 (0.511–1.279)	
Tumor size				
≤ T2	89 (67.7%)	136 (69.0%)	1.00	*p =* 0.384
> T2	49 (32.3%)	61 (31.0%)	0.815 (0.514–1.292)	
Lymph node metastasis				
No	137 (99.3%)	189 (95.9%)	1.00	*p =* 0.099
Yes	1 (0.7%)	8 (4.1%)	5.799 (0.717–46.902)	
Distant metastasis				
No	136 (98.6%)	183 (92.9%)	1.00	*p* = 0.031*
Yes	2 (1.4%)	14 (7.1%)	5.202 (1.163–23.270)	
Vascular invasion				
No	110 (79.7%)	167 (84.8%)	1.00	*p =* 0.230
Yes	28 (20.3%)	30 (15.2%)	0.706 (0.400–1.246)	
Child-Pugh grade				
A	103 (74.6%)	157 (79.7%)	1.00	*p =* 0.275
B or C	35 (25.4%)	40 (20.3%)	0.750 (0.447–1.258)	
HBsAg				
Negative	83 (60.1%)	112 (56.9%)	1.00	*p =* 0.548
Positive	55 (39.9%)	85 (43.2%)	1.145 (0.736–1.782)	
Anti-HCV				
Negative	78 (56.5%)	103 (52.3%)	1.00	*p =* 0.444
Positive	60 (43.5%)	94 (47.7%)	1.186 (0. 766–1.838)	
Liver cirrhosis				
Negative	29 (21.0%)	32 (16.2%)	1.00	*p =* 0.267
Positive	109 (79.0%)	165 (83.8%)	1.372 (0.785–2.396)	

The ORs with analyzed by their 95% CIs were estimated by logistic regression models.

> T2: multiple tumor more than 5 cm or tumor involving a major branch of the portal or hepatic vein(s).

*p value < 0.05 as statistically significant.

**Table 4:** Odds ratio (OR) and 95% confidence interval (CI) of clinical status and *MTNR1A* rs2375801 genotypic frequencies in 335 HCC patients

Variable	Genotypic frequencies			
	AA (*N* = 110)	AG+GG (*N* = 225)	OR (95% CI)	*p* value
Clinical Stage				
Stage I/II	68 (61.8%)	155 (68.9%)	1.00	*p =* 0.198
Stage III/IV	42 (38.2%)	70 (31.1%)	0.731 (0.454-1.178)	
Tumor size				
≤ T2	68 (61.8%)	157 (69.8%)	1.00	*p =* 0.146
> T2	42 (38.2%)	68 (30.2%)	0.701 (0. 435-1.131)	
Lymph node metastasis				
No	109 (99.1%)	217 (96.4%)	1.00	*p =* 0.192
Yes	1 (0.9%)	8 (3.6%)	4.018 (0.496-32.539)	
Distant metastasis				
No	109 (99.1%)	210 (93.3%)	1.00	*p* = 0.048*
Yes	1 (0.9%)	15 (6.7%)	7.782 (1.015-59.663)	
Vascular invasion				
No	87 (79.1%)	190 (84.4%)	1.00	*p =* 0.225
Yes	23 (20.9%)	35 (15.6%)	0.697 (0.389-1.249)	
Child-Pugh grade				
A	85 (77.3%)	175 (77.8%)	1.00	*p =* 0.917
B or C	25 (22.7%)	50 (22.2%)	0.971 (0.563-1.676)	
HBsAg				
Negative	69 (62.7%)	126 (56.0%)	1.00	*p =* 0.242
Positive	41 (37.3%)	99 (44.0%)	1.322 (0.828-2.111)	
Anti-HCV				
Negative	61 (55.5%)	120 (53.3%)	1.00	*p =* 0.715
Positive	49 (44.5%)	105 (46.7%)	1.089 (0.689-1.722)	
Liver cirrhosis				
Negative	27 (24.6%)	34 (15.1%)	1.00	*p* = 0.037*
Positive	83 (75.4%)	191 (84.9%)	1.827 (1.036-3.222)	

The ORs with analyzed by their 95% CIs were estimated by logistic regression models.

> T2: multiple tumor more than 5 cm or tumor involving a major branch of the portal or hepatic vein(s).

*p value < 0.05 as statistically significant.

We also investigated the possible association between the melatonin receptor gene polymorphisms and the levels of serological markers of HCC, including α-fetoprotein (AFP), alanine transaminase **(***ALT***)**, and aspartate transaminase (AST). As a consequence, we found that the serum levels of AST in HCC patients who possess at least one polymorphic allele of *MTNR1A* rs6553010 were elevated as compared with that from those who are homozygous for the reference allele (Table 5). In addition, the levels of AFP in patients who are positive for the minor allele of *MTNR1B* rs10830963 (heterozygotes or homozygotes) were higher than that of HCC cases negative for this variant (Table 5).

**Table 5:** Association of *MTNR1A-MTNR1B* genotypic frequencies with the HCC laboratory findings

Characteristic	α-Fetoprotein^a^ (ng/mL)	AST (IU/L)	ALT (IU/L)	AST/ALT ratio
**rs13140012**				
AA	429.0 ± 163.0	45.58 ± 4.02	44.04 ± 3.38	1.19 ± 0.03
AT+TT	722.3 ± 262.0	50.39 ± 5.21	45.63 ± 4.17	1.21 ± 0.03
*p* value	0.342	0.465	0.767	0.561
**rs6553010**				
AA	546.6 ± 239.7	41.19 ± 3.13	39.77 ± 2.53	1.18 ± 0.02
AG+GG	650.6 ± 237.5	54.44 ± 5.84	49.30 ± 4.73	1.22 ± 0.03
*p* value	0.758	0.046*	0.076	0.333
**rs2119882**				
TT	288.0 ± 102.7	44.23 ± 3.87	42.12 ± 3.26	1.21 ± 0.03
TC+CC	818.3 ± 275.6	51.31 ± 5.26	46.94 ± 4.22	1.20 ± 0.03
p value	0.072	0.279	0.366	0.769
**rs13113549**				
GG	411.1 ± 164.5	45.87 ± 4.11	43.83 ± 3.46	1.21 ± 0.03
GA+AA	730.3 ± 259.0	50.14 ± 5.14	45.75 ± 4.12	1.20 ± 0.03
*p* value	0.298	0.517	0.721	0.892
**rs2375801**				
AA	334.3 ± 124.8	43.26 ± 4.03	40.25 ± 3.02	1.20 ± 0.03
AG+GG	738.4 ± 246.2	51.04 ± 4.85	47.36 ± 3.98	1.20 ± 0.03
p value	0.144	0.217	0.155	0.862
**rs1387153**				
CC	480.1 ± 224.0	54.69 ± 9.43	49.66 ± 6.41	1.19 ± 0.04
CT+TT	653.3 ± 219.8	45.93 ± 3.11	43.10 ± 3.02	1.21 ± 0.02
*p* value	0.581	0.378	0.355	0.683
**rs1562444**				
AA	513.6 ± 198.6	43.66 ± 2.86	40.14 ± 2.61	1.23 ± 0.04
AG+GG	684.6 ± 267.6	52.76 ± 6.13	49.36 ± 4.85	1.18 ± 0.02
*p* value	0.608	0.179	0.094	0.254
**rs4611171**				
GG	512.3 ± 195.5	43.59 ± 2.83	39.83 ± 2.57	1.23 ± 0.03
GT+TT	688.4 ± 271.6	52.96 ± 6.22	49.79 ± 4.92	1.18 ± 0.02
*p* value	0.599	0.171	0.073	0.159
**rs10765576**				
GG	504.8 ± 193.6	48.33 ± 4.54	41.94 ± 2.87	1.24 ± 0.04
GA+ AA	697.1 ± 274.0	48.55 ± 5.29	47.88 ± 4.80	1.17 ± 0.02
*p* value	0.567	0.975	0.289	0.102
**rs10830963**				
CC	233.6 ± 89.5	55.41 ± 9.24	47.05 ± 5.97	1.22 ± 0.04
CG+GG	763.9 ± 239.4	45.42 ± 3.02	44.09 ± 3.13	1.19 ± 0.02
*p* value	0.038*	0.304	0.661	0.539

Mann-Whitney *U* test was used between two groups.

a Mean ± S.E.

*p value < 0.05 as statistically significant.

### Association of MTNR1A and MTNR1B haplotypes with HCC

**Figure 1:**
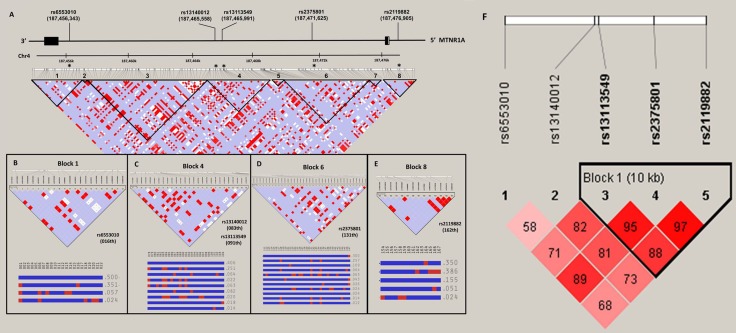
MTNR1A gene structure and linkage disequilibrium map constructed from 167 selected SNP tags (**A**) In the LD map, SNP-pairwise correlation coefficients and Hedrick’s multi-allelic D’) in the East Asian population (HCB+JPT, 1000 genome project) are shown in black squares when D’= 1.0 and white squares when D’ = 0. The “Four Gamete” conventional grey scale is used to display the LDs shown in black through grey (the colour intensity decreases with the decrease in the D’ value) generated by Haploviewversion 4.2. SNPs located within and around the MTNR1Agene were plotted against the chromosomal positions Chr.4: 187,454,000 to 187,478,000 (GRCh37.p13, genome assembly). Total 2 exons in this gene (NM_005958.3), the five selected SNPs, rs6553010, rs13140012, rs13113549, rs2375801 and rs2119882. Codon exons, introns and untranslated regions are indicated with filled boxes, thin lines and unfilled boxes from the 3′-to 5′-end of this gene (reverse strain), respectively. The black stars indicate the selected genotyping of the MTNR1ASNP polymorphisms. The five selected SNPs as an alleles of that region with recombination rate plotted and haplotypes display, (**B**) rs6553010(016th), (**C**) rs13140012(083th) and rs13113549 (091th) (**D**) rs2375801 (131th) and (**E**) rs2119882 (162th), locate on LD map of haplotype block 1, 4, 6 and 8, respectively. (**F**) Linkage disequilibrium (LD) map for single nucleotide polymorphisms in the MTNR1A gene in this study. Block is pairwise *D*’ plots and haplotype blocks obtained from HAPLOVIEW.

**Figure 2:**
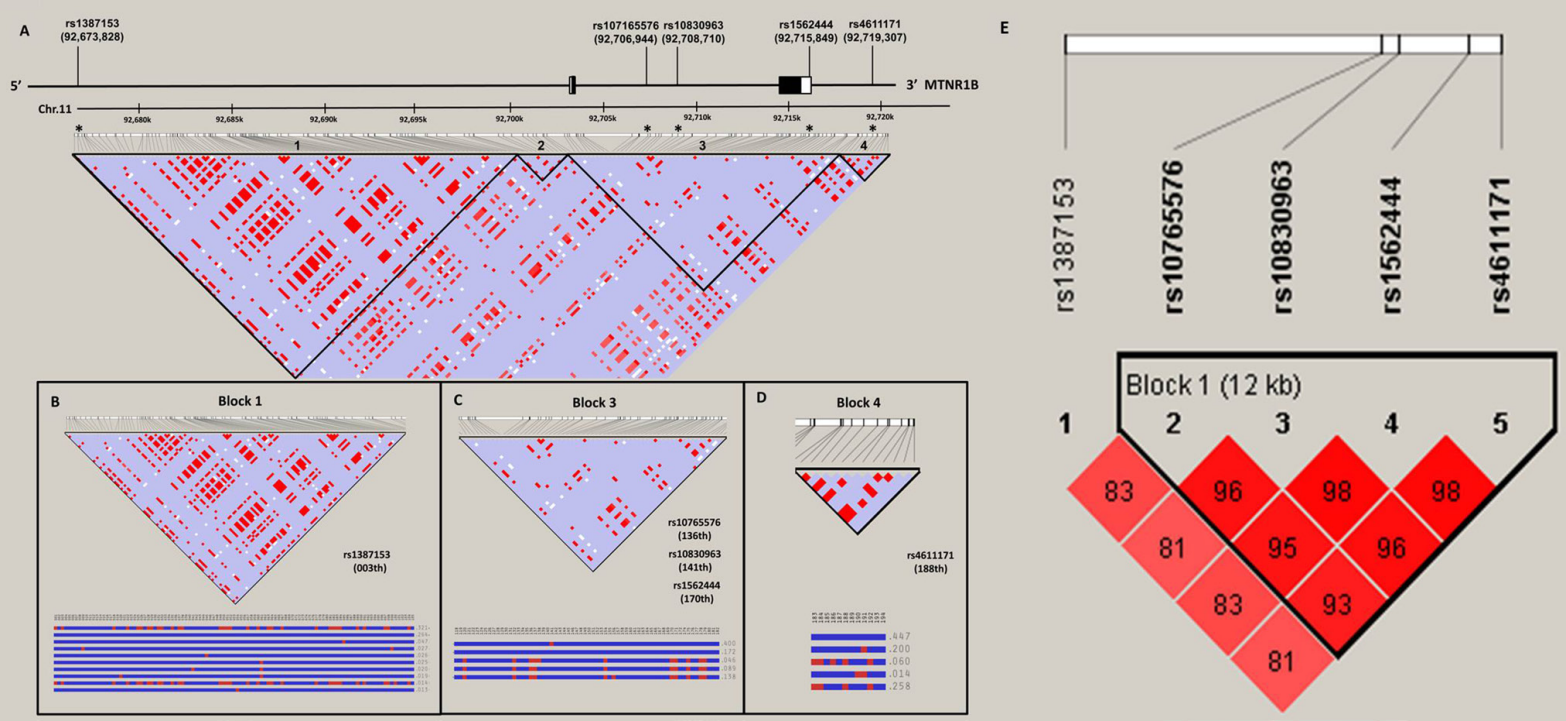
MTNR1B gene structure and linkage disequilibrium map constructed from 194 selected SNP tags (**A**) In the LD map, SNP-pairwise correlation coefficients and Hedrick’s multi-allelic D’) in the East Asian population (HCB+JPT, 1000 genome project) are shown in black squares when D’ = 1.0 and white squares when D’ = 0. The “Four Gamete” conventional grey scale is used to display the LDs shown in black through grey (the colour intensity decreases with the decrease in the D’ value) generated by Haploviewversion 4.2. SNPs located within and around the MTNR1Bgene were plotted against the chromosomal positions Chr.11: 92,673,000 to 92,720,000 (GRCh37.p13, genome assembly). Total 2 exons in this gene (NM_005959.3), the five selected SNPs, rs1387153, rs10765576, rs10830963, rs1562444 and rs4611171. Codon exons, introns and untranslated regions are indicated with filled boxes, thin lines and unfilled boxes from the 5′-to 3′-end of this gene, respectively. The black stars indicate the selected genotyping of the MTNR1BSNP polymorphisms. The five selected SNPs as an alleles of that region with recombination rate plotted and haplotypes display, (**B**) rs1387153 (003th), (**C**) rs10765576 (136th), rs10830963 (141th) and rs1562444 (170th) and (**D**) rs4611171 (188th), located on LD map of haplotype block 1, 3 and 4, respectively. (**E**) Linkage disequilibrium (LD) map for single nucleotide polymorphisms in the MTNR1B gene in this study. Block is pairwise *D*’ plots and haplotype blocks obtained from HAPLOVIEW.

The relationship of melatonin receptor gene haplotypes with the risk of developing HCC was also assessed. Two haplotype blocks containing sets of SNPs within the *MTNR1A* (Figure 1) and *MTNR1B* gene (Figure 2) were defined by the use of pairwise disequilibrium for SNPs evaluated in this study [30]. The distributions of haplotype frequencies are shown in Tables 6 and 7, with the most frequent haplotype in the controls (GAT for *MTNR1A* and GGAG for *MTNR1B*) being chosen as the reference. While adjusted for age and alcohol use, we found that a specific haplotype of *MTNR1B* (GCGT) was significantly associated with increased susceptibility to HCC (AOR, 2.132; 95% CI, 1.106–4.111; *p =* 0.024), further suggesting a genetic predisposition of *MTNR1B* to liver cancer.

**Table 6:** Frequencies of *MTNR1A* haplotypes in HCC patients and control subjects

Haplotype block			Controls	Patients	
rs13113549 G/A	rs2375801 A/G	rs2119882 T/C	*n* = 2392	*n* = 670	AOR (95% CI)^a^
G	A	T	1378 (57.6%)	384 (57.3%)	1.000 (reference)
A	G	C	795 (33.1%)	214 (31.9%)	1.000 (0.821–1.218)
G	G	T	81 (3.4%)	22 (3.3%)	1.084 (0.652–1.803)
A	G	T	59 (2.5%)	23 (3.4%)	1.259 (0.741–2.137)
G	G	C	52 (2.2%)	20 (3.0%)	1.543 (0.887–2.683)
A	A	T	16 (0.7%)	5 (0.8%)	1.153 (0.396–3.362)
G	A	C	5 (0.2%)	2 (0.3%)	1.617 (0.282–9.258)
A	A	C	6 (0.3%)	0 (0.0%)	−

^a^Adjusting for the effects of age and alcohol drinking.

**Table 7:** Frequencies of *MTNR1B* haplotypes in HCC patients and control subjects

Haplotype block				Controls	Patients	
rs10765576 G/A	rs10830963 C/G	rs1562444 A/G	rs4611171 G/T	*n* = 2392	*n* = 670	AOR (95% CI) ^a^
G	G	A	G	1050 (43.9%)	293 (43.7%)	1.000 (reference)
A	C	G	T	697 (29.1%)	181 (27.0%)	0.950 (0.763–1.181)
G	C	A	G	577 (24.1%)	152 (22.7%)	0.979 (0.777–1.234)
G	C	G	T	28 (1.2%)	17 (2.5%)	2.132 (1.106–4.111) ^c^
A	C	A	G	11 (0.5%)	5 (0.8%)	1.674 (0.533–5.257)
A	C	G	G	10 (0.4%)	7 (1.0%)	2.355 (0.861–6.440)
A	G	A	G	5 (0.2%)	5 (0.8%)	2.722 (0.697–10.629)
Others^b^				14 (0.6%)	10 (1.5%)	2.283 (0.974–5.349)

^a^Adjusting for the effects of age and alcohol drinking.

^b^Others: GCGG (7), GGAT (5), GCAT (5), GGGT (3), ACAT (2), and AGGT (2).

^c^*p* = 0.024.

### Functional analysis of the MTNR1A rs2119882 locus

**Figure 3:**
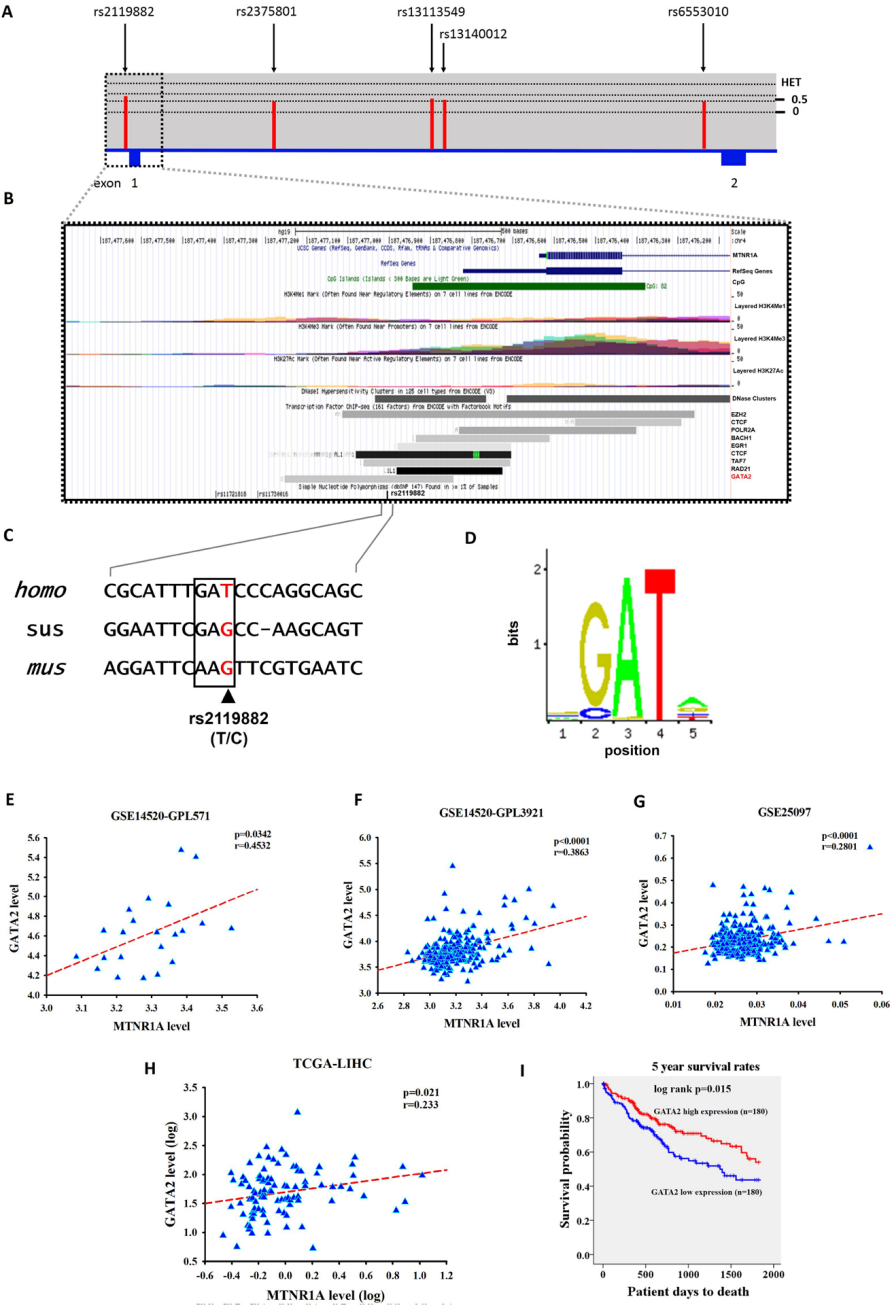
Gene features and SNPs (rs2119882) analyzed of human MTNR1A (NM_005958.4) (**A**) Exons are revealed by the filled blue and are numbered 1 and 2 from chromosome positions chr4: 187,454, 809 to 187, 476, 537 (GRCh37.p13). The above panel shows population-specific heterozygosity frequencies of the polymorphism in the East Asian population. SNPs were indicated by black arrows and labelled with the SNP ID numbers. (**B**) Expanded view of ENCODE database for the promoter region containing rs2119882 using UCSC genome browser. The CpG, H3K4Me1, H3K4Me3, and H3K27Ac tracks which are predictive chromatin signatures in the human genome, as determined by the ChIP-seq assays. DNase cluster tracks show DNase hypersensitivity areas. Transcription factors tracks shown the region of transcription factor binding sites derived from a large collection of ChIP-seq experiments performed by ENDCODE project. (**C**) Upstream sequence in the MTNR1A transcripts of human (*homo*, NM_005958.4), pig (*sus*, XM_013985006.1), and mouse (*mus*, NM_008639.2) sequences shown in this alignment. Consensus residues of GATA2 transcription factor binding sites are above this alignment in box and rs2119882 SNP revealed in color red. (**D**) DNA motif logo of the GATA2 (MA0036.1) consensus sequence from the JASPAR CORE database. (**E**) The correlation between GATA2 and MTNR1A expression in the hepatocellular carcinoma specimens in publicly available microarray datasets from the GSE14520-GPL571 project. Pearson’s correlation coefficient = 0.4532; *p =* 0.0342. (**F**) The correlation between GATA2 and MTNR1A expression in the hepatocellular carcinoma specimens in publicly available microarray datasets from the GSE14520-GPL3921 project. Pearson’s correlation coefficient = 0.3863; *p <* 0.0001. (**G**) The correlation between GATA2 and MTNR1A expression in the hepatocellular carcinoma specimens in publicly available microarray datasets from the GSE25097 project. Pearson’s correlation coefficient = 0.2801; *p <* 0.0001. (**H**) The correlation between GATA2 and MTNR1A expression in the hepatocellular carcinoma specimens from The Cancer Genome Atlas (TCGA) Data. Pearson’s correlation coefficient = 0.233; *p =* 0.021. (**I**) An overall survival curve was produced for patients with high (red lines) and low (blue lines) GATA2 mRNA expression levels using the Kaplan--Meier method. The *p* values were determined using a log-rank test.

As a preliminary assessment of the putative functional role of these SNPs, we investigated the possible function of *MTNR1A* rs2119882. As shown in Figure 3A, rs2119882 is located within the promoter region of *MTNR1A* gene, which is enriched with the putative binding site of many transcription factors (Figures 3B–3D). Among these transcription factors, levels of GATA2, a GATA-binding protein whose expression has been relevant to the invasion and migration of HCC cells [31], were found to be correlated with MT_1_ expression in the publicly available microarray datasets and TCGA dataset (Figure 3E–3H). Moreover, as shown in Figure 3I, the results from the TCGA dataset of hepatocellular carcinoma patients indicated that low GATA2 expression was associated with a poor overall survival. These data suggest that *MTNR1A* variants may alter melatonin receptor 1A expression likely through perturbing the binding of transcription factors to its promoter and subsequently contribute to promotion of not only tumor cell growth but also invasion.

## DISCUSSION

The development of hepatocarcinogenesis is a complex process that is modulated by both environmental and genetic factors. In the present study, we showed that melatonin receptor gene polymorphisms were associated with the increased incidence, metastasis, and hepatocellular damage in HCC, revealing for the first time a genetic predisposition of melatonin receptors to liver cancer.

Manipulation of melatonin signaling has been linked to the regulation of different hallmarks of cancer mainly through activation of MT_1_ and MT_2_ [17–19, 29, 32, 33]. Multiple mechanisms have been suggested for the inhibitory effects of melatonin on cancer development and progression. One notable example is its actions on energy metabolism. Melatonin appears to signal via its high-affinity receptors to modulate glucose homeostasis by alterations in insulin release and leptin production [34, 35]. It has been demonstrated that *MTNR1B* gene polymorphisms are associated with features of metabolic syndrome, including diabetes [21–24]. Some cancer types tend to develop more frequently in patients with diabetes, as the relative risks imparted by diabetes are greatest (about two-fold or higher) for tumors of the liver [36]. In addition, diabetes-related factors including steatosis, nonalcoholic fatty liver disease (NAFLD), and cirrhosis may also enhance susceptibility to liver cancer. In our investigation, we found that a specific haplotype of *MTNR1B* was significantly associated with increased susceptibility to HCC (Table 7) and that HCC patients who carry the variant allele of *MTNR1B* SNP (rs10830963) exhibited higher levels of AFP than did cases negative for this variant (Table 5). Other than pancreatic cells, melatonin also modulates energy balance via its direct effects on adipocytes through activation of MT_1_ [37]. Altered functions of adipose tissue are central to metabolic syndrome and NAFLD as well [38]. A significant association of *MTNR1A* gene polymorphism (rs2119882) with another comorbidity of metabolic syndrome, polycystic ovary syndrome has been reported [26]. Here, we observed that HCC patients positive for at least one polymorphic allele of *MTNR1A* rs2119882 (Table 3) or of its linked SNP rs2375801 (Table 4) were more prone to develop distant metastasis. Additionally, a relationship between *MTNR1A* SNP rs6553010 and increased occurrence of HCC was revealed in the present study (Table 2).

In addition to energy metabolism, two mechanisms implicated in the oncostatic properties of MT_1_ and MT_2_ are modulation of cancer cell proliferation/induction of apoptosis and activation of the immune system [33]. The anti-proliferative effects of melatonin on cancer are largely mediated by the activation of MT_1_ [39–42], but an action of MT_2_ cannot be excluded [43]. In term of serving as a regulator of the immune system in tumor prevention, both MT_1_ and MT_2_ are expressed in a wide variety of immune cells and contribute to inhibition of the formation of virus-induced tumors by eliminating viral infections (HCC is commonly associated with HBV and HCV infection) and identification and destruction of tumor cells that express tumor-specific antigens [44–47]. Specifically, engagement of melatonin with MT_1_ or MT_2_ allows induction of cytokine secretion, modulation of lymphocyte functions, restoration of impaired activity of T-helper cells in immune-depressed conditions, promotion of T-lymphocyte proliferation, inhibition of precursor B-cell apoptosis in the bone marrow, and protection of CD4+ T cells from apoptosis. These findings, together with our data suggest that variants of melatonin receptors may alter cancer cell growth and activation of immune responses, elevating the risk for hepatic tumors.

We observed here in a correlation of *MTNR1A* gene polymorphisms (rs2119882 and rs2375801) with increased risk of distant metastasis in HCC patients (Tables 3 and 4). Cancer metastasis, which accounts for most deaths due to malignancies, is a multistage process that requires cancer cells to escape from the primary site, survive in the circulation, and develop in distant tissues [48]. A broad range of melatonin’s actions on counteracting metastases has been documented [18, 19]. In melanoma and breast cancer, it is demonstrated that the level and function of MT_1_ were correlated with cancer cell invasion and metastasis [49, 50]. Intriguingly, rs2119882 is located on the putative binding site of many transcription factors within the promoter region of *MTNR1A* gene. Among these transcription factors, GATA2 expression levels were correlated with MT_1_ expression in the TCGA dataset. Furthermore, attenuated expression of GATA2 promotes migration and invasion of HCC cells *in vitro* and be associated with poor prognosis of liver cancer [31]. Peters et al. [51] also reported that GATA2 is involved in clear cell renal cell carcinoma tumor development and aggressiveness. These results suggest that rs2119882 polymorphism may alter the binding affinity of transcription factors to the promoter of *MTNR1A* gene, leading to changes in MT_1_ levels and subsequent impacts on tumor metastasis.

Our data identify an impact of *MTNR1A/MTNR1B* gene variations on the risk and progression of HCC; however, additional work is needed to address several limitations of the present study. One is that the high level of heterogeneity, in term of the severity and subtype of liver cancer or the diversity of HCC-related clinical manifestations, such as diabetes, NAFLD, HBV, and HCV infection, within the case cohort may validly give rise to different conclusions regarding the relationships between melatonin receptor gene polymorphisms and development of HCC. Another weakness is that the effects of acquired risks on the susceptibility of liver cancer may be underestimtated due to a lack of population stratification based on the amount or duration of alcohol use. In addition, the genetic association observed in this study might be restricted to particular ethnic groups unless replication studies are carried out.

Taken together, our results show that SNP rs6553010 of *MTNR1A* and a particular haplotype of *MTNR1B* causally contribute to an increased risk of HCC. In addition, an association of *MTNR1A* gene polymorphisms (rs2119882 and rs2375801) was detected with the occurrence of distant metastasis in HCC. These findings highlight a novel genetic predisposition to liver tumorigenesis.

## MATERIALS AND METHODS

### Subjects

This study included 335 patients with HCC and 1196 cancer-free controls; it was approved by the institutional review board of Chung Shan Medical University Hospital in Taichung, Taiwan. For the control group, we selected 1196 healthy individuals with no self-reported history of cancer at any site from Taiwan Biobank. The diagnoses of HCC were verified histologically in all cases. HCC patients were staged clinically at the time of diagnosis according to the TNM staging system of the American Joint Committee on Cancer (AJCC) [52]. Liver cirrhosis was diagnosed by liver biopsy, abdominal sonography, or biochemical evidence of liver parenchymal damage with endoscopic esophageal or gastric varices. Clinicopathological parameters, including clinical staging, tumor size, lymph node metastasis, distant metastasis, presence of HBV surface antigen (HBsAg), reactivity with antibody against HCV (anti-HCV), liver cirrhosis, the levels of α-fetoprotein (AFP), aspartate aminotransferase (AST) and alanine aminotransferase (ALT), were obtained from chart reviews. During the same study period, 1196 ethnically matched individuals who have neither diagnosed with HCC nor self-reported history of cancer of any sites were enrolled as controls.

### Demographic data

Information regarding age, gender, alcohol consumption, and tobacco use was obtained from each participant. Alcohol consumption is defined as having up to an average of more than 2 drinks per day. Tobacco use is defined as current smoking of at least one cigarette per day during the latest three months.

### Genotyping

Genomic DNA was extracted using QIAamp DNA blood mini kits (Qiagen, Valencia, CA, USA). Allelic discriminations of five *MTNR1A* SNPs [ rs13140012 (Assay ID: C_31861431_10), rs6553010 (Assay ID: C_11782809_10), rs2119882 (Assay ID: C_16100974_10), rs13113549 (Assay ID: C_11791559_10), and rs2375801 (Assay ID: C_15785462_10)] and five *MTNR1B* SNPs [rs1387153 (Assay ID: C_1932612_10), rs1562444 (Assay ID: C_8369474_10), rs4611171 (Assay ID: C_3256853_20), rs10765576 (Assay ID: C_30851514_10), and rs10830963 (Assay ID: C_3256858_10)] were assessed by using the TaqMan assay with an ABI StepOne™ Real-Time PCR System (Applied Biosystems, Foster City, CA, USA), and analyzed with SDS version 3.0 software (Applied Biosystems, Foster City, CA, USA).

### Bioinformatics analysis

We proposed several standard protocol bioinformatics tools for assessing where polymorphic rs2119882 genetic variants were associated with a putative function that might influence patient outcomes. The predictive chromatin signatures in the human genome, as determined by the ChIP-seq assays from the Encyclopedia of DNA Elements (ENCODE) project [53]. UCSC Cancer Genomics Browser [54] for hepatocellular adenocarcinomas were used for analyzing MTNR1A expression, DNA methylation, molecular features, and clinical outcomes. The potential consensus GATA2 cis-regulatory DNA elements through JASPAR [55] CORE collection (http://jaspar.genereg.net).

### Statistical analysis

The Hardy-Weinberg equilibrium was assessed by using a goodness-of-fit v2 test for biallelic markers. Mann–Whitney *U* test and Fisher’s exact test were used to compare the differences in demographic characteristics between healthy controls and HCC patients. The adjusted odds ratios (AORs) with their 95% confidence intervals (CIs) for the association between genotype frequencies and the risk of HCC plus clinicopathological characteristics were estimated by multiple logistic regression models after controlling for other covariates. The haplotype-based analysis was conducted using the Phase program [56]. A *p* value < 0.05 was considered significant. The data were analyzed using SAS statistical software (Version 9.1, 2005; SAS Institute Inc., Cary, NC).
